# Saphenous vein graft pseudoaneurysm: living with a ticking bomb

**DOI:** 10.1093/ehjcr/ytag534

**Published:** 2026-07-17

**Authors:** Stergios Soulaidopoulos, Fotis Tatakis, Eirini Dri, Konstantinos P Tsioufis

**Affiliations:** First Cardiology Department, Hippokration Hospital, Athens Medical School, School of Medicine ‘Hippokration’ General Hospital, National and Kapodistrian University of Athens, 114 Vasilissis Sofias avenue, Athens 11527, Greece; First Cardiology Department, Hippokration Hospital, Athens Medical School, School of Medicine ‘Hippokration’ General Hospital, National and Kapodistrian University of Athens, 114 Vasilissis Sofias avenue, Athens 11527, Greece; First Cardiology Department, Hippokration Hospital, Athens Medical School, School of Medicine ‘Hippokration’ General Hospital, National and Kapodistrian University of Athens, 114 Vasilissis Sofias avenue, Athens 11527, Greece; First Cardiology Department, Hippokration Hospital, Athens Medical School, School of Medicine ‘Hippokration’ General Hospital, National and Kapodistrian University of Athens, 114 Vasilissis Sofias avenue, Athens 11527, Greece

**Keywords:** Saphenous vein graft, Pseudoaneurysm, Coronary artery bypass graft, Coronary artery disease, Coronary angiography

## Case description

**Figure 1:**
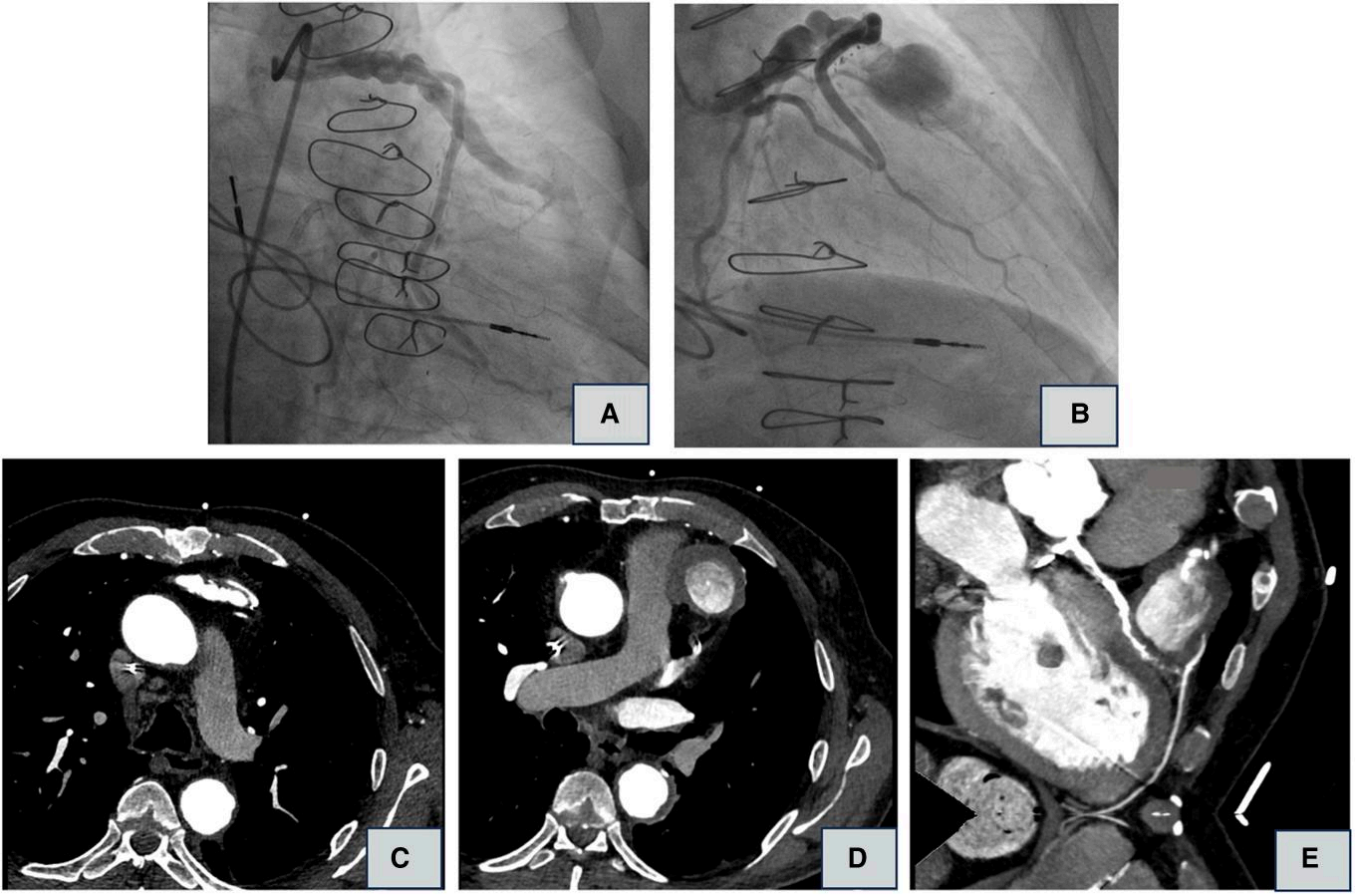
(*A*) Aneurysmatic dilatation of the saphenous vein graft (SVG) to the diagonal branch as presented in the coronary angiography performed 3 years ago. (*B*) Substantial extravasation of contrast medium from the SVG into a giant circular aneurysmatic formation as shown in the new coronary angiography (corresponding dynamic sequence available in Supplementary material online, *Video S1*) (*C*—*E*) Computed tomography demonstrating a 43 × 47mm pseudoaneurysm in front of the anterior left ventricular wall, adjacent to the left anterior descending artery and the left internal mammary artery, perfused by the aneurysmatic SVG (corresponding imaging sequence available in Supplementary material online, *Video S2*).

A 67-year-old male with a history of quadruple coronary artery bypass grafting (CABG) 10 years prior underwent coronary angiography for exertional angina. Compared to a coronary angiography performed 3 years earlier, which had shown only moderate aneurysmatic dilation of the saphenous vein graft (SVG) to the diagonal branch (*Figure 1A*), the current procedure revealed that the lesion had rapidly progressed into a large circular aneurysmatic formation with contrast extravasation in the midportion of the SVG to the diagonal branch (*Figure 1B*, Supplementary material online, *Video S1*). Regarding the other grafts, a patent left internal mammary artery (LIMA) to the left anterior descending artery (LAD), a patent SVG to the obtuse marginal branch, and an occluded SVG to the right coronary artery were also observed.

A computed tomography angiography was subsequently performed, which confirmed the presence of a 43×37 mm pseudoaneurysm (*Figure 1 C-E*, Supplementary material online, *Video S2*). While small or stable SGG aneurysms can occasionally be managed conservatively or via percutaneous approaches, such as covered stent deployment or coil embolization, considering the high risk of rupture and the presence of ischaemic symptoms in this case, a multidisciplinary team recommended surgical intervention, and the pseudoaneurysm was successfully resected.

Pseudoaneurysms of SVGs are a rare complication following CABG, occurring in less than 1% of patients.^1^ They may be detected incidentally during coronary angiography in asymptomatic individuals or present with symptoms such as chest pain, dyspnoea, syncope, or haemoptysis.

In contrast to arterial grafts, in which aneurysm formation is extremely rare, SVGs are more prone to progressive degenerative changes after implantation, reflected by higher rates of graft occlusion and aneurysmal dilatation. This phenomenon is largely attributed to the structural mismatch and the relatively thin wall of vein conduits exposed to systemic arterial pressures, as well as their tendency towards accelerated atherosclerotic degeneration, occasionally compounded by technical factors during graft preparation.^2^ Larger pseudoaneurysms carry significant risks of thrombosis, distal embolization, or rupture and therefore frequently require surgical or percutaneous intervention, while management decisions should be guided by rupture risk, patient comorbidities, and the need to maintain adequate myocardial revascularization.

While SVG pseudoaneurysms are documented in the literature, this case is uniquely valuable because it provides a clear, longitudinal visual record over a 3-year window. By directly contrasting the baseline mild angiographic dilation with the rapid progression into a massive pseudoaneurysm, it highlights the unpredictable nature of vein conduit degeneration and serves as a strong clinical reminder to maintain a low threshold for timely Heart Team intervention before catastrophic rupture occurs.

## Supplementary Material

ytag534_Supplementary_Data

## Data Availability

All data supporting the findings of this case report are included within the main manuscript file and the accompanying online supplementary data.

## Notes

**Consent:** The authors confirm that written consent for submission and publication of this case report, including images and associated text, has been obtained from the patient.
